# The Night Medical Service

**Published:** 1880-08

**Authors:** 


THE NIGHT MEDICAL SERVICE.—We are glad to learn from the *Medical Record* that the plan recommended by Dr. Nachtel of Paris, in his paper read before the Academy of Medicine of New York, has become a law in that metropolis. It is the system of night service in Paris, adapted to the wants of the latter city. The law provides, "that there shall be kept at each police station a list of physicians approved by the Health Board, who shall be ready to respond to night calls, for the sum of three dollars per capita." In case the patient is too poor to pay for the service, a certificate of attendance is issued by the officer having charge of the station, which certificate is duly cashed by the treasurer of the service fund. There can be no reasonable doubt but that Baltimore would be greatly benefited by the enactment of a similar law, and as such we would respectfully urge upon our municipal authorities the propriety of taking steps towards having such a law here. Should this be done, and the suggestions made, in a previous issue of this journal, for supplying physicians and medicines, in certain districts into which the city should be divided, be adopted, we believe we would have the best regulated system for promptly meeting the wants of our people, rich and poor, day and night, with professional assistance, to be found anywhere; if so, let us set the example for the benefit of all other communities, as early as possible.

